# 3D deep convolution neural network for radiation pneumonitis prediction following stereotactic body radiotherapy

**DOI:** 10.1002/acm2.13875

**Published:** 2022-12-22

**Authors:** Rishabh Kapoor, William Sleeman, Jatinder Palta, Elisabeth Weiss

**Affiliations:** ^1^ Department of Radiation Oncology Virginia Commonwealth University Richmond Virginia USA

**Keywords:** artificial intelligence, deep learning, DenseNet 121, outcome prediction, radiation pneumonitis, ResNet‐50

## Abstract

In this study, we investigated 3D convolutional neural networks (CNNs) with input from radiographic and dosimetric datasets of primary lung tumors and surrounding lung volumes to predict the likelihood of radiation pneumonitis (RP). Pre‐treatment, 3‐ and 6‐month follow‐up computed tomography (CT) and 3D dose datasets from one hundred and ninety‐three NSCLC patients treated with stereotactic body radiotherapy (SBRT) were retrospectively collected and analyzed for this study. DenseNet‐121 and ResNet‐50 models were selected for this study as they are deep neural networks and have been proven to have high accuracy for complex image classification tasks. Both were modified with 3D convolution and max pooling layers to accept 3D datasets. We used a minority class oversampling approach and data augmentation to address the challenges of data imbalance and data scarcity. We built two sets of models for classification of three (No RP, Grade 1 RP, Grade 2 RP) and two (No RP, Yes RP) classes as outputs. The 3D DenseNet‐121 models performed better (F1 score [0.81], AUC [0.91] [three class]; F1 score [0.77], AUC [0.84] [two class]) than the 3D ResNet‐50 models (F1 score [0.54], AUC [0.72] [three‐class]; F1 score [0.68], AUC [0.71] [two‐class]) (*p* = 0.017 for three class predictions). We also attempted to identify salient regions within the input 3D image dataset via integrated gradient (IG) techniques to assess the relevance of the tumor surrounding volume for RP stratification. These techniques appeared to indicate the significance of the tumor and surrounding regions in the prediction of RP. Overall, 3D CNNs performed well to predict clinical RP in our cohort based on the provided image sets and radiotherapy dose information.

## 1 INTRODUCTION

Stereotactic body radiotherapy (SBRT) is the standard of care for medically inoperable patients with early‐stage NSCLC resulting in excellent local control and typically low treatment‐related morbidity.^1^, ^2^ Among the most common complications observed with SBRT are radiation pneumonitis (RP) and pulmonary fibrosis. Due to the smaller treatment volumes, the incidence of RP is less than in locally advanced lung cancers and is generally observed in ≤10% of patients after up to 6 months from treatment completion.^3^ Despite its lower incidence, RP is a serious side effect with potentially lethal outcomes in this population with typically severely compromised lung function. Radiographic signs of RP are observed with CT images that indicate ground‐glass opacities and patchy or confluent consolidations in the lung tissue.^4^ These imaging characteristics are seen within 3–6 months post‐treatment.^5^ The diagnosis of RP is often subjective and is typically based on clinical evaluation and radiological findings. While several risk factors associated with RP have been identified, such as dose‐ and volume‐dependent factors^3^ or interstitial lung disease,^6^ the prediction of the individual RP risk is difficult and complex.

Various studies have been performed to assess lung density changes on CT as a metric of parenchymal lung changes after conventional radiotherapy (RT) and SBRT. Dose‐dependent increases in regional lung density with conventional RT^7^ and SBRT^8^, ^9^ were identified, but no quantitative correlation has been established for predicting RP. Furthermore, for several pneumonitis patients, no major lung density changes have been observed.^10^ Studies have also shown the average increase in lung density is related to the percent reduction in pulmonary function tests indicating functional lung changes.^11^ Lung density changes, location of the tumor (upper vs. lower lobe), total lung volume, and radiation field design are some of the attributes that contribute to radiation‐induced toxicities.^12^ There is a need for multiple higher‐order pattern recognition metrics and techniques that can capture and model the intricacies of these toxicity patterns.

Deep learning approaches have shown great promise in the medical imaging domain with image‐recognition tasks where intricate biological interactions are extracted more effectively without defining these features manually. As opposed to the subjective visual assessment of images by trained physicians and extracting engineered features such as radiomics, these deep learning methods automatically identify and quantitatively evaluate complex patterns in the dataset and select the most robust features. Deep learning methods have performed better than their traditional statistical counterparts in many imaging tasks such as multimodality image registration,^13^ automatic contouring,^14^ and survival analysis.^15^ Convolutional neural networks (CNNs) are a class of deep learning methods that combine different sizes of imaging filters with a network of neurons through a series of interconnected linear and non‐linear layers. As part of the training, the CNN image filters learn high‐ and low‐level imaging features, eventually making predictions on the desired outputs. Li et al. first applied a 3D CNN model for the evaluation of treatment response in locally advanced esophageal squamous cell carcinoma.^16^ Ibragimov et al. applied CNNs to 3D dose distributions of the rectum surface for toxicity predictions.^17^ With a few exceptions, most of these studies lack generalization of their models and results due to insufficient data – under 100 patients. Many of these studies have used 2D data for their efforts or alternatively, used 3D datasets with a limited volume in and around the tumor region only. It is important to note that none of these methods have been utilized as a part of the clinical routine yet. Since there are very few published and shared 3D CNN models that have been trained on medical or general images, there are no medical‐to‐medical transfer learning approaches being applied to solve similar imaging classification problems until now.

In this study, we investigate 3D CNNs with the input of radiographic and dosimetric characteristics in the lung tumor and surrounding lung volume to predict the likelihood of RP for NSCLC patients treated with SBRT. Reliable prediction of pneumonitis risk may guide individualized treatment approaches and reduce pulmonary toxicity. We designed an analytical setup with a dataset of NSCLC patients imaged before radiotherapy (pre‐treatment imaging), and 3 and 6 months after treatment (post‐treatment imaging), to discover the prognostic power of CNNs as a binary RP classification problem.

## 2 METHODS

### 2.1 Dataset

This study includes a total of 193 primary lung cancer patients treated with SBRT from 2008 to 2020 at our facility. Following approval by the institutional review committee, clinical data, radiographic images, and dose distribution matrices were extracted from the medical record, PACS, and the treatment planning system to create a database for analysis. Only patients who had a follow‐up visit with CT scans at 3 and 6 months were included in this study. Table 1 details the clinical and dose prescription characteristics of the patients included in this study. The internal gross tumor volume (iGTV) was delineated on the maximum intensity projection (MIP) images from the 4D CT (Brilliance Big Bore, Philips, Amsterdam, Netherlands) dataset, or on a single‐phase image and propagated over all phases of the 4D CT. For patients treated in breath hold, an iGTV was created based on 3 repeat breath‐hold CTs. The pixel spacing for these images is 0.98–1.37 mm with a slice thickness of 3 mm, in‐plane matrix size of 512 × 512 acquired with 120–140 kVP. The planning target volume (PTV) was generated by adding an expansion margin of typically 5 mm. The dose covering 95% of the PTV volume was 102.5 ± 4.2% of the prescription dose. Image‐guidance using cone beam CT was applied to align the target daily prior to treatment delivery. RP was clinically evaluated at 3‐ and 6‐month follow‐up visits based on clinical symptoms and radiographic findings.

**TABLE 1:** Patient characteristics and treatment regimen

Clinical characteristics		*P*‐value
Staging (no of patient)	IA (*n* = 147) IB (*n* = 27) IIA (*n* = 10) IIB (*n* = 2) IIIA (*n* = 3) IV (*n* = 4)	0.58
Gender	Male (*n* = 99) Female (*n* = 94)	0.22
Median age (range)	69.2 ± 10.4 years	0.54
Karnofsky score (%)	80 ± 10	0.90
PTV volume	36.05 ± 29.6 cc	0.17
Prescription dose (Gy)/fractions (no of patients)	48 Gy/4 (*n* = 142) 50 Gy/5 (*n* = 26) 40 Gy/5 (*n* = 12) 60 Gy/5 (*n* = 6) 60 Gy/8 (*n* = 5) 45 Gy/5 (*n* = 2)	0.11
RP status (number of patients)	No RP [RP0] (*n* = 154) RP Grade 1 [RP1] (*n* = 26) RP Grade > = 2 [RP2] (*n* = 13)	

### 2.2 Imaging and treatment planning dataset

The pre‐treatment images used for treatment planning comprised of either an end inspiration CT scan for patients treated with breath hold or the 30% phase or average image set of a 4D CT scan. Post‐treatment imaging was performed with comfortable inspiration breath hold on diagnostic CT scanners at 3‐ and 6‐month intervals after the completion of the radiation treatment. These CT scans were acquired typically with end inspiration scanning techniques. All thoracic CT scans for follow‐up visits were acquired on either GE or Siemens CT scanners and reconstructed with sharp kernels. A total of 579 3D CT images and 193 dose datasets were analyzed during this study.

### 2.3 Image registration

**FIGURE 1:**
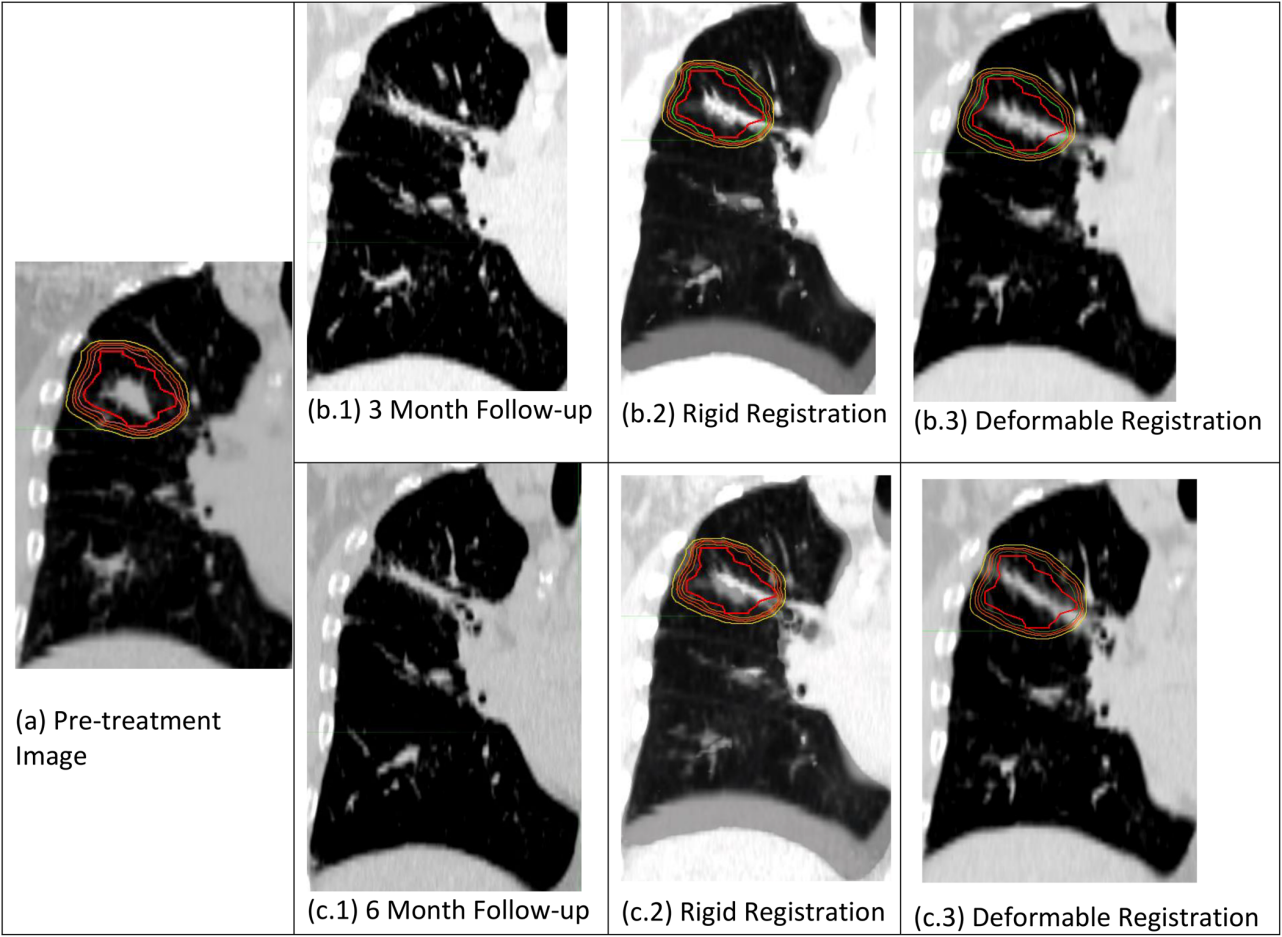
Example of image registration. (a) Baseline pre‐treatment (used for treatment planning) CT scan (coronal section) with the PTV (red) and isodose lines. (b.1) 3‐month follow‐up CT scan. (b.2) Rigid registration with pre‐treatment CT scan and 3 month follow‐CT scan. (b.3) Deformable registration with pre‐treatment CT scan and 3‐month follow‐up CT scan with PTV volume and isodose curves. (c.1) 6‐month follow‐up CT scan. (c.2) Rigid registration with pre‐treatment CT scan and 6 month follow‐CT scan. (c.3) Deformable registration with pre‐treatment CT scan and 6‐month follow‐up CT scan with PTV volume and isodose curves

To account for changes in the anatomy between the CT scans used at treatment planning (pre‐treatment) and follow‐up, deformable image registration was performed. These radiographic changes were due to post‐treatment volume loss, distortion, fibrosis, and tumor regression and had an impact on the overall lung architecture. Because follow‐up images were high‐quality images and essentially free of artifacts, follow‐up CTs could be registered directly to planning CTs (either inspiration BH or average images from 4D CTs). The follow‐up CT scans were first rigidly registered to the baseline CT scan used for treatment planning. This step aligned the two 3D datasets slice by slice using a clinically utilized image registration software (MIM Maestro version 7.0). Visual inspection of the automatic rigid registration was performed with manual adjustments to the translation and rotation parameters using a box‐shaped mask to align the spine and vertebral structures. In the next step, the deformable registration algorithm from MIM Maestro was utilized. To avoid the algorithm to perform non‐physiological and non‐realistic deformations, radiographic lung changes and PTV regions of the follow‐up CT and planning CT were given a value of −250 HU prior to the registration for the algorithm to avoid overfitting to small anatomical discrepancies. The corresponding 120 cm^3^ regions on longitudinal image sets were co‐registered to minimize potential differences in the alignment of patient anatomy while preserving radiographic lung changes due to radiotherapy All registrations were individually verified based on the overlay of the two image sets and matching the regions of interest (ROIs) such as lung tissue, PTV, chest wall, airways, trachea, etc. The rigid and deformable registrations were repeatedly performed until a visually acceptable solution was found for each dataset (Figure 1). In the final step, the radiotherapy 3D dose distribution (RT Dose) and contours (RT Structure Set) were mapped onto the follow‐up CT scans. All datasets including the registered CT, RT Dose, RT Plan (for planning dataset only), and RT Structure Set were exported in DICOM format and stored in a folder.

### 2.4 Data preprocessing for deep learning

All imaging, RT Dose and RT Structure Set files were imported in a custom‐built software coded in python v3.7.13. All datasets were resampled into isotropic voxels of unit dimensions to ensure comparability, where 1‐unit voxel is equal to 1 mm.^3^ These interpolations were carried out using the nearest neighbor interpolation methods for images, dose, and contour datasets. Using the full 3D tumor and lung volume contours from the RT Structure Sets, both the center of mass and the bounding box of the PTV was computed. Using this center of mass and bounding box, 3D isotropic patches of size 120 × 120 × 120 were extracted from the imaging and dose datasets. These 3D patch extractions were manually verified and shifted in three dimensions to maximize the capture of the lung and PTV volume within the patch size. The 3D dose patches for each dataset were overlaid on the imaging patches to visually verify image‐dose registrations. The imaging patches were normalized to a 0–1 range using the upper and lower HU bounds (−1024 to 2048). The dose patches were normalized to a 0–1 range using 0–60 Gy dose range and any value greater than 60 Gy was normalized to 1. The high‐density regions outside the lung volume such as bone tissue were patched out of the input samples since these tend to be non‐informative and can potentially confound the deep learning models.

**FIGURE 2:**
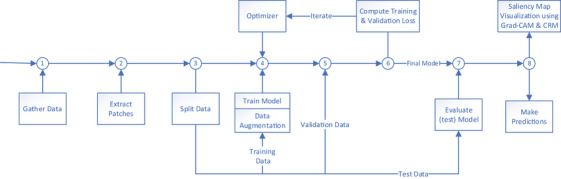
The study design of the proposed model for radiation pneumonitis (RP) prediction classification

Due to the fact that there is a class imbalance in our dataset where the number of patients (154 patients or 79.8%) who did not indicate radiation pneumonitis toxicity (no RP) greatly outnumber the patients who showed pneumonitis symptoms (26 patients – grade 1 [13%] [RP1], 13 patients – grade > = 2 [6.5%] [RP2]), data augmentation techniques were applied to randomly oversample^18^ the patches with the minority class (RP1 and RP2), yielding the training size of 1182 input samples. This technique created new input samples for training without actually altering the visual characteristics of the images. These augmentations included random flipping of the 3D image and dose patches along the left‐right and superior‐inferior axes, random translations ± 10 voxels in three‐dimensional space, and random rotations of 5, 7, and 10 degrees along the longitudinal axes. These class‐specific perturbations were applied to the initial test set to make our DL models robust and to reduce bias and generalization errors. These basic data augmentation techniques have been the most popular approach for recent medical imaging research.^19^ With the help of these augmentation and oversampling techniques, we created an equal number of image and dose samples for the three classes used in the prediction models. This technique may also help with overfitting as it produces new patches with similar properties as the original data but also fills previously unoccupied feature space. Furthermore, similar image augmentation techniques were utilized in real‐time during training for the purpose of avoiding overfitting and making the models generalizable. The total input samples were defined in an 80:20 ratio in training and testing input samples. The testing samples were not exposed to the training process. The training input samples were further split in an 80:20 ratio between the final training and validation set. The split was stratified by the three prediction classes, which ensured that an equal percentage of data is taken from each class for training. Figure 2 shows the study design. The models were trained on the training dataset and used to predict the test dataset to evaluate the model performance. Once the model was trained and validated, we tested the model with the testing input samples (unseen by the model during training).

### 2.5 Deep learning

Traditionally, deep learning for medical imaging datasets has been confined to 2D CNNs where predictions are made on a per slice basis in two‐dimensional space, and then final predictions are obtained through highest probability, voting, or other methods based on the prediction results from all slices in the dataset. These methods work fine with 2D medical images such as computed radiography (CRs) and x‐rays. With three‐dimensional images, the algorithm loses the contextual information of the shape, size, and texture of the lesions, tumor, and organs between the slices of images, and hence the prediction performance is low. We used the 3D CNN models for this study which are very much like 2D CNNs except that it uses 3D convolution and max pooling layers.

**FIGURE 3:**
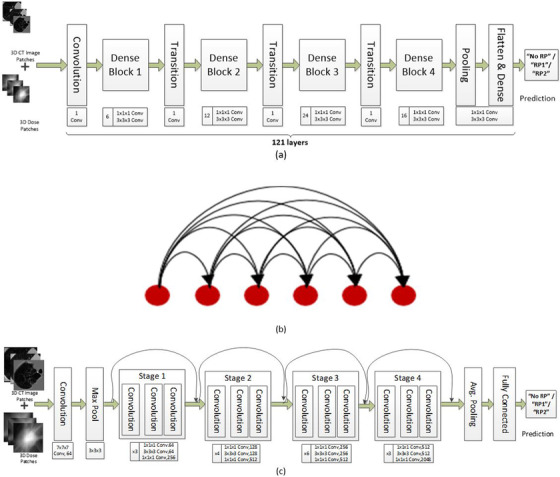
(a) DenseNet 121 – 3D architecture: A deep DenseNet with four dense blocks. The transition layers in between the successive dense blocks are responsible to change the feature‐map sizes via convolution and pooling operations. This architecture has 121 layers with interconnected layers in a feed forward fashion to ensure maximum information flow between layers in the network. (b) The connections between the 121‐layer blocks of the DenseNet‐121 CNN network where there are direct connections from any layer to all subsequent layers. The connection between different layer blocks increases variation in the input of subsequent layers via feature reuse and improves efficiency. With this architecture the vanishing gradient and loss problems are resolved since each layer has direct access to the gradients from the loss function and the original input signal, leading to an implicit deep supervision. (c) ResNet‐50 – 3D architecture: A residual network of 50 parameter layers where the subtraction of features is learned from the input of that layer by using shortcut connections which is shown as curved arrow

For our study, we modified the published 2D CNN models, namely DenseNet‐121 and ResNet‐50.^20^, ^21^ These models use deeper neural networks which are generally more difficult to train. These models have the residual learning framework to ease the training of networks that are substantially deeper than those used with basic general 2D images such as VGG‐16^22^ etc. Using substantially deeper networks has been shown to be more accurate and more efficient to train if they contain shorter connections between the layers that are closer to the input and closer to the output layer.^23^ For our models, we modified the 2D convolution kernels of the 2D basic blocks to a 3D convolution kernel and built a 3D basic block. The basic architecture (Figure 3a) and structure (Figure 3b) of the DenseNet‐121 and ResNet‐50 (Figure 3c) were unchanged. With modification of the 3D basic blocks, we built 3D DenseNet‐121 and 3D ResNet‐50 models. With traditional deep learning models, there is a tendency for the prediction accuracy to decrease as the depth of the layers increases beyond a certain number. This problem was solved by passing features from the lower layers to the higher layers thus eliminating the vanishing gradient problem. Here all the features learned by the first few layers can be utilized throughout all the subsequent layers thus reducing the number of trainable parameters (Figure 3a). The ResNet‐50 model is built to train deeper CNNs by creating shortcuts (skip connections) between the front and back layers. DenseNet‐121 is built with the same concept but it establishes dense connections of all the previous and subsequent layers. It has been reported that DenseNet achieves comparable performance to ResNet‐50 with fewer parameters and less computation costs.^24^

The input channels for these models were the 120 × 120 × 120 cube image patches and the same sized 3D dose patch as an additional input channel and the output were either three classes (No RP, RP1, RP2) or two classes (No RP, Yes RP) with the purpose to perform binary classification of these input patches. Since we were performing a binary classification task, the output layer of the network is a dense layer whose activation function is sigmoid, and the output values range between 0 and 1. Since CNN models are typically trained with millions of image sets and due to be unavailability of such a dataset for our study, we utilized the publicly available lung image database consortium (LIDC) and Image database resource initiative (IDRI)^25^ data set to shallowly train our CNN models. These datasets are being utilized to train CNN models for lung nodule detection and are comprised of 1000 lung CT images. We trained our model for 20 epochs with a binary classification output layer with the purpose to initialize the convolution filter within our model to recognize the presence of the lung lesion in the input datasets. The IDRI dataset was utilized to initialize and pre‐train the model weights before utilizing the model for actual training with our CT and dose datasets. Training details are as follows: we used the gradient‐based stochastic optimizer called Adam with a learning rate of 0.001 with a decay rate of 0.96 and decay step of 1 × 10^4^, a batch size of 4, and trained both models for 100 epochs with a total of 11.3 M trainable parameters for DenseNet‐121 and 46.2 M trainable parameters for ResNet‐50 models. In order to avoid overfitting, we utilized early stopping techniques where the training monitored the loss function and stopped training with a patience value (the number of epochs to wait before early stop if no progress on the validation set) of 10. Our model was trained multiple times with an early stopping value of 2–20. We observed that a stopping value of more than 10 showed that the model loss was constant, and accuracy was showing minor three decimal places improvements indicating overfitting. We also included batch normalization layers in order to improve convergence and generalization in the models. TensorFlow 2.8 was used to train and test the CNN. Google's web‐based coding note called Collaboratory, or Collab, was used to execute code on Google's cloud‐based NVIDIA Tesla P100 GPU servers with 25.46GB available memory size.

### 2.6 Visual explanation of convolution neural network predictions: Explainable AI – integrated gradients

Explainable AI is a research field in machine learning interpretability techniques whose aim is to understand machine learning model predictions and explain them in human understandable terms to build trust with stakeholders. In computer vision, a saliency map is an image that can be in the form of a heat map that shows each input voxel's unique quality with the goal to represent the image and the predictions from the DL models into something that is more meaningful and easier to analyze. These heat maps predominately help explain how a model made its decision on a particular dataset although these explanations are not guaranteed to make sense to human experts and these explanations are also not considered to follow any known rules or decision trees that are traditionally used for image classification algorithms. These heat map techniques do not make the CNN model interpretable where accuracy and human understandable relationships could be derived between the inputs and the output of the CNN models. Our purpose with using these saliency heat map techniques was to qualitatively review the model and gather more insights into model predictions. This is clearly a developmental goal where we are testing if these maps can be usefully created for our 3D datasets and understand the general areas in the image where the network is focused to make the predictions. Explainability and interpretability of the deep learning model is a topic of research^26^ and with this work, we are trying to use industry‐standard techniques that have been successfully utilized to highlight areas in real‐world non‐medical photographic images for CNN model predictions. Here we are briefly describing the integrated gradient (IG) methods that are used to localize and highlight important ROIs for a particular category within an input image set with the purpose to explain the CNN model predictions. The feature attribution heat maps help to highlight frequently missed features in the 3D imaging datasets thus complementing human judgment by physicians with accurately grading the dataset.

### 2.7 Integrated gradients (IG)

To visualize the highlighted map of the most important features in an input image set with respect to predictions made, we utilized the integrated gradient method (IG).^27^ The computation of gradients of the model output with respect to the input features provides an analog for feature importance. IG^28^ uses the gradient information of a target class flowing back into the last convolution layer to form visual heat maps from the CNN‐based DL models. This method provides pixel‐based maps that measure the contribution of each pixel in the input image to a predicted pneumonitis class. The contributions are measured relative to a baseline image, which is intended to provide no information to the model. For this study, we used a black image as the baseline. We verified that our trained algorithm predicted no prediction for this baseline. For each 3D image‐set, we generated a path of 100 steps, in which each step was interpolated between the blank baseline image‐set and the target image‐set. For each output pneumonitis class, we summed model gradients over each pixel and took the absolute value. We then surfaced the heatmap for the pneumonitis class with the highest score.

### 2.8 Evaluation metrics

The dataset used in this work is highly imbalanced with a smaller number of samples with an RP status than the non‐RP status. The metrics used to evaluate the performance of these models need to be agnostic to the data imbalance. Since we are evaluating a multi‐class problem, we have used the macro‐averaged metrics instead micro‐averaged ones. The overall performance of a multi‐class classifier is commonly obtained by taking an average of individual class performances. The advantage of using these metrics is that micro‐average assigns equal weight to each sample (or instance), whereas macro‐average assigns equal weight to each type (or class), and with highly imbalanced datasets, reporting micro‐averaged performance would be misleading because the class with fewer samples (i.e., rare class) are given less importance than the class with more samples. With macro‐averaged metrics, equal importance is given to all classes irrespective of the number of samples in each class.

The expressions for the macro‐averaged metrics are as follows.

(1) $$Precison_{macro}=\frac{1}{N}\;\sum_{c=1}^{N}\frac{TP_{c}}{TP_{c}+FP_{c}}$$

(2) $$Recall_{macro}=\frac{1}{N}\;\sum_{c=1}^{N}\frac{TP_{c}}{TP_{c}+FN_{c}}$$

(3) $$F_{1}\;Score_{macro}=\frac{1}{N}\;\sum_{c=1}^{N}2.\frac{Precision_{c}-Recall_{c}}{Precision_{c}+Recall_{c}}$$

(4) $$Accuracy=\frac{TP+TN}{TP+TN+FP+FN}$$

TP (true positive): when the model predicted as positive, and the ground truth is positive (e.g., an ‘RP1’ sample is identified as ‘RP1’ by the model).

TN (true negative): when the model predicts as negative, and the ground truth is negative (e.g., an ‘RP1’ sample is not identified as a ‘No RP’ sample by the model).

FP (false positive) (Type I error): when the model predicted as positive, but ground truth is negative (e.g., the model predicts the sample to the ‘RP1’ class, but the ground truth is false).

FN (false negative) (Type II error): when the model predicted as negative, but the ground truth is positive (e.g., the model does not predict the sample to the ‘RP1’ class, but the ground truth is true).

Accuracy is the proportion of correct predictions over the total number of samples. Recall (sensitivity) is defined by calculating the total predicted positives out of the total number of actual positives. Precision (positive predictive value) is defined by calculating the total number of actual positive samples out of predicted positive samples. F1 score is the harmonic average of the precision and recall values and is a widely used evaluation measure for a classification problem.

We also used the confusion matrices to assess the model performance. The correct/incorrect number of predictions are shown by the count values which are further divided in individual classes. A confusion matrix contains information about the model's confusion in predicting between classes and performance of a model. The performance of the models was also assessed via the areas under the receiver operating characteristic curve (AUCs). Finally, the AUCs of the two models (DenseNet‐121 and ResNet‐50) were compared by the Mann–Whitney U test with the Bonferroni correction. Two‐sided *p* < 0.05 were considered to indicate statistical significance.

## 3 RESULTS

### 3.1 Clinical characteristics

The patients’ clinical and dosimetric characteristics are provided in Table 1. The patients had follow‐up visits with the treating radiation oncologist 3 months following the completion of radiation treatment and subsequently every 3 months thereafter. Clinical RP was evaluated and scored by the treating oncologists as part of the patient's assessments according to the common terminology toxicity criteria (CTCAE) version 5.^29^ Our cohort is quite homogeneous (95% have stage I–IIA, there are no major variations is performance status, 93% were treated with BED ≥100 (as generally recommended). We do not expect to find any imbalance in our dataset based on this homogeneity. The only factors that vary and might influence RP risk are PTV size and dose, both of which are already inherently included in the models. Patients with higher stages had either oligometastatic disease with a small primary lung cancer and isolated metastases outside the chest or had lung cancers in separate lobes where one of the tumors was resected. Fractionation schedules were selected based on tumor size and location. *P*‐values are computed from the chi‐square test of independence to determine the significant association between the specific variable listed in Table 1 and RP status. Since none of the *p*‐value are less than 0.05, we fail to reject the null hypothesis. This means we do not have sufficient evidence to say that there is an association between the clinical characteristics and RP status.

### 3.2 Prediction performance of the 3D DenseNet‐121 model

**FIGURE 4:**
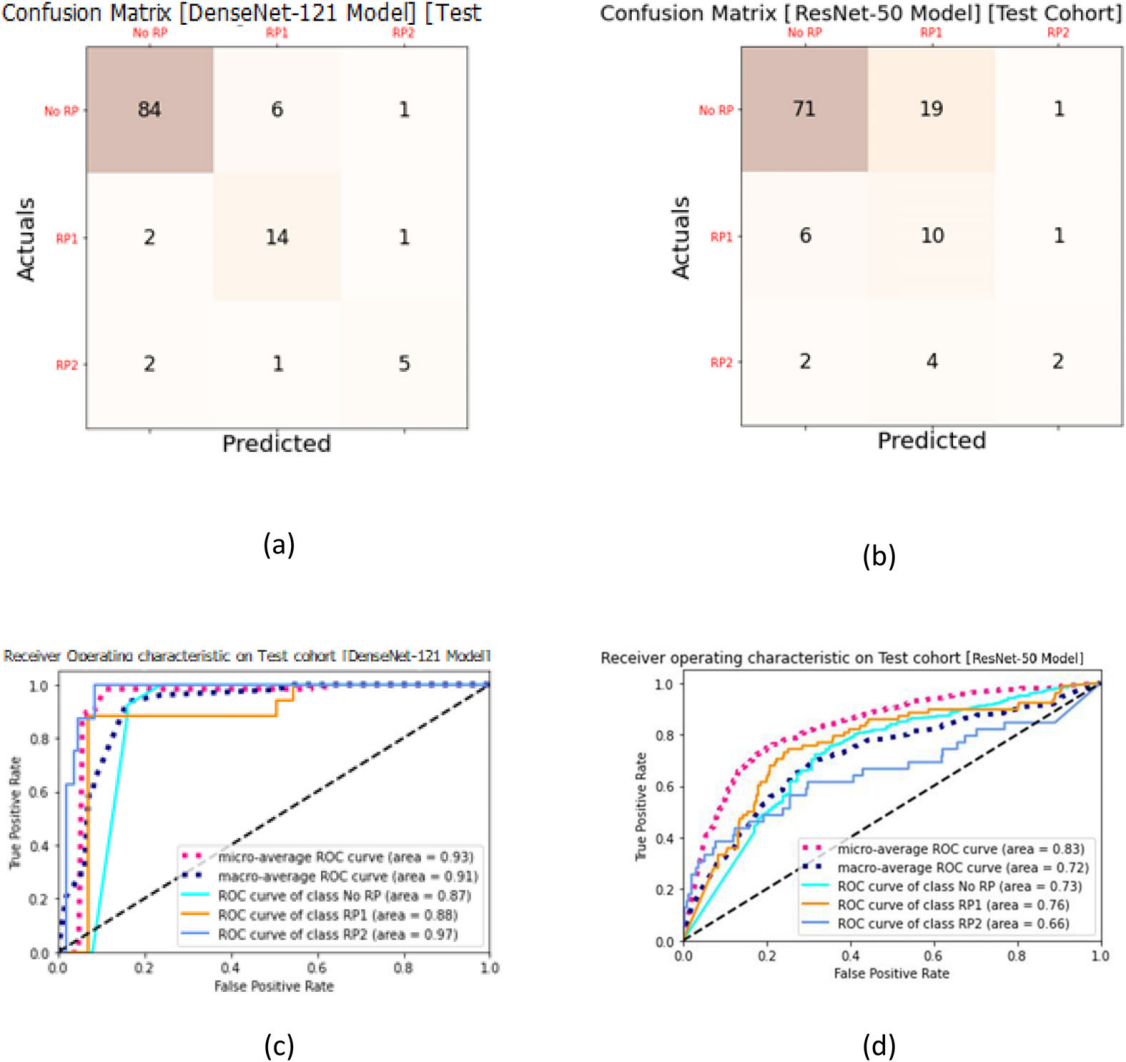
Evaluation of the 3D Dense‐121 versus ResNet‐50 model trained with 3D image + 3D dose patches from the pre‐treatment and follow‐up datasets. (a) Confusion matrix for three class prediction with the 20% sample set (test set) that was not seen or trained on the DenseNet‐121 model. (b) Confusion matrix for the ResNet‐50. Darker color cells demonstrate more accurate predictions, and the diagonal shows the labels predicted correctly. (c) Prognostic power (ROC) and true positive rate versus false positive rate curves derived from the test set for Dense‐121 model. (d) Same chart for the ResNet‐50 model

**FIGURE 5:**
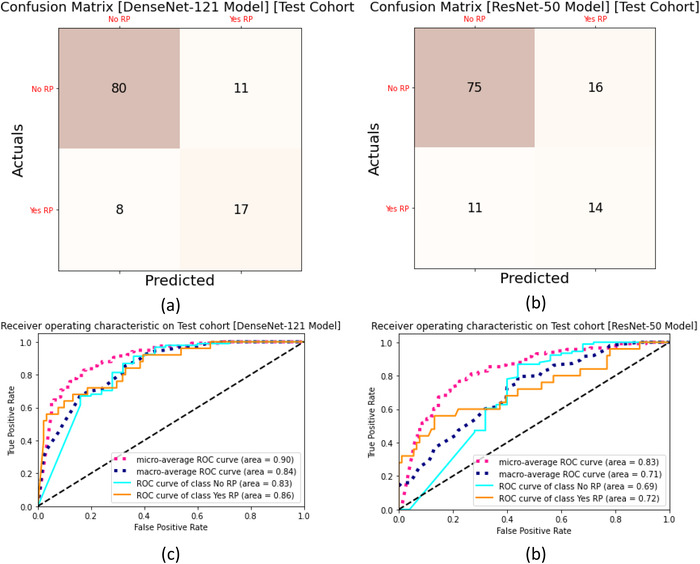
Evaluation of the 3D Dense‐121 versus ResNet‐50 model trained with 3D image + 3D dose patches for the two‐class prediction

In assessing the ability of CNN models to quantify radiographic traits and characteristics of the lung and tumor region, we performed an analysis based on the test dataset that was never exposed or seen by the model during training. In this section, we present the results and prediction evaluation of our models. Here we made two copies each for the 3D DenseNet‐121 and ResNet‐50 models, the first two models were trained to predict three classes (No RP [RP0], RP Grade 1 [RP1], RP Grade > = 2 [RP2]) and the next two models were trained to predict two classes (No RP, Yes RP [including all grades]). All models were trained with 80% of pre‐treatment and follow‐up CT datasets with the 3D dose patches and tested using the unseen remaining 20% of the dataset. All models were independently trained, validated, and tested. Figure 4 shows the confusion matrix and ROC and true positive rate versus false positive rate (precision vs. recall) curves for the 3D DenseNet‐121 and ResNet‐50 models for the three‐class prediction. For three class prediction, the DenseNet‐121 model had an AUC_macro_ = 0.91 and F1 score_macro_ = 0.81, whereas the ResNet‐50 model had an AUC_macro_ = 0.72 and F1 score_macro_ = 0.54 (Table 2). Mann–Whitney U test performed for pair‐wise comparison of AUCs among the two model types had a *p*‐value of 0.017 indicating that the three‐class DenseNet‐121 showed significantly better performance than the ResNet‐50 model. Figure 4 shows the confusion matrix, ROC, and true positive rate versus false positive rate (precision vs. recall) curves for the 3D DenseNet‐121 and ResNet‐50 models. The test dataset contains 116 data samples, 91 of which are ‘RP0’ samples, 17 were ‘RP1’ samples and 8 were ‘RP2’ samples. From the confusion matrix shown in Figure 4, the DenseNet‐121 model can accurately identify 84 ‘RP0’, 14 ‘RP1’, and 8 ‘RP2’ samples for three class predictions. In total, this model could accurately identify a total of 103 data samples (88%). The ResNet‐50 model could accurately identify a total of 83 data samples (72%). For two class prediction, the DenseNet‐121 model had an AUC_macro_ = 0.84 and F1 score_macro_ = 0.77, whereas the ResNet‐50 model had an AUC_macro_ = 0.71 and F1 score_macro_ = 0.68 (Table 2). Mann–Whitney U test performed for pair‐wise comparison of AUCs among the two model types had a *p*‐value of 0.527. Figure 5 shows the confusion matrix, ROC, and true positive rate versus false positive rate (precision vs. recall) curves for the 3D DenseNet‐121 and ResNet‐50 model. The test dataset contains 116 data samples, 91 of which are ‘No RP’ samples and 25 of which are ‘Yes RP’ samples. From the confusion matrix shown in Figure 5, the DenseNet‐121 model can accurately identify 80 ‘No RP’ and 17 ‘Yes RP1’ samples. In total, this model could accurately identify a total of 97 data samples (83%). The ResNet‐50 model could accurately identify a total of 89 data samples (77%). Since these models showed higher accuracy than prediction models that utilize dose volume histogram and dose function histogram^30^ based prediction models (AUC = 0.73) using support vector machine techniques, logistic regression classifiers^31^ (AUC values 0.64–0.75), we are confident in the DenseNet‐121 model's ability to accurately assess whether a patient would have RP or not, even when predicting new, unseen patients.

**TABLE 2:** Macro‐averaged precision, recall, F1 score, and overall accuracy for the four models based on the test cohort (not seen or trained on the model) and training cohorts

Training dataset	Prediction classes	Testing dataset	Model	Precision_macro_	Recall_macro_	F1 score_macro_	Accuracy	AUC_macro_	*P*‐value
Pre‐treatment and follow‐up dataset [80% split]	Three class prediction	Test cohort (20% split)	DenseNet‐121	0.84	0.78	0.81	0.89	0.91	0.017
			ResNet‐50	0.57	0.59	0.54	0.72	0.72	
	Two class prediction	Test cohort (20% split)	DenseNet‐121	0.76	0.78	0.77	0.84	0.84	0.527
			ResNet‐50	0.67	0.69	0.68	0.77	0.71	

### 3.3 Localization evaluation

**FIGURE 6:**
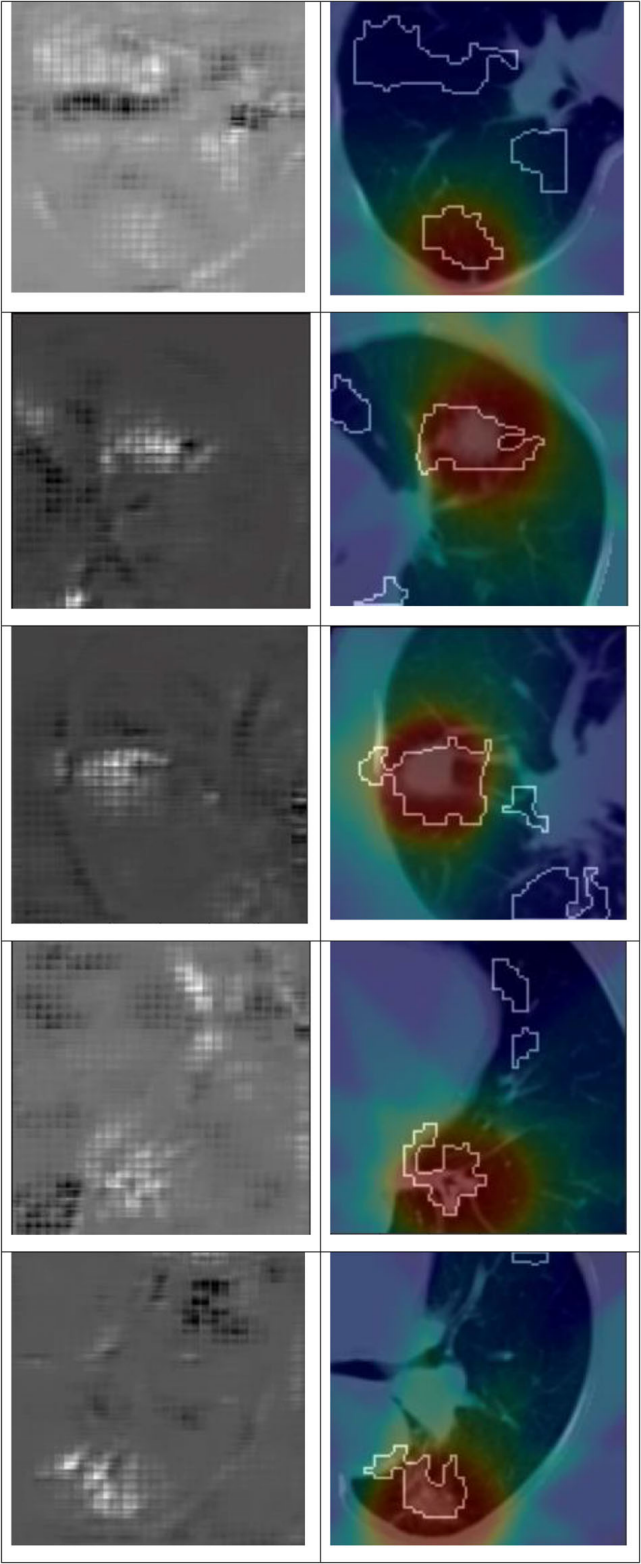
Visual display of the most important areas of the input 3D dataset that have the most contributions to maximize the outputs of the final prediction layer used to predict a RP case. The rows represent five different patient samples (axial slice) that encompass the PTV volume. The first column displays the integrated gradient (IG) heat maps. Bright (white) regions represent positive gradients, and dark (black) regions show negative gradients. The second column represents the CT patch annotated dose maps (displayed as heat map) and contours of voxel regions that are in top 50% of IG maps

We also analyzed the 3D DenseNet‐121 model built to predict the three classes by rendering the IG heat maps that provide information on the importance of each region and voxel relative to the final prediction class. The IG map can help understand how the model scores the input data with learned features (e.g., dose distribution pattern, imaging features) from different regions of the input. Generally, regions of the IG map with bright regions correspond to regions whose learned features are more important for RP prediction. We observed that the network highlighted the regions of the tumor and its interface with the parenchyma or pleura regions of the lung which shows that these regions are critical for the model in discriminating toxicity from non‐toxicities. Based on our qualitative analysis of thirty RP1 + RP2 patient cases, these heat maps agreed with the tumor and dose regions and their surrounding voxels and also included some voxels outside the tumor or dose regions, as shown in Figure. 6. Relatively higher density regions that included the tumor and the surrounding regions had the most contributions to the predictions. In some patients, the heat maps showed a rather focused area, whereas in other patient cases, these heat maps were rather widespread covering large parts of the lung. None of these models aim at providing physiological modeling using the tissue type characteristics of the tumor and lung structure and are using the information present in the image textures and patterns to pick out unique traits and attributes that are used in the eventual classification. As such, while showing the high dose and tumor area as important for RP prediction is reasonable from the clinical perspective, identification of other lung areas as relevant for RP through the IG process cannot be reasonably verified based on the current understanding of RP development. Examples of the IG heat maps are shown in Figure 6 for five patient cases who had grade 2 RP.

## 4 DISCUSSION

This is one of the first studies to predict post‐SBRT pneumonitis using a comparatively large CT dataset. We demonstrated the ability of CNN models to significantly stratify patients into none versus grade 1 versus grade 2 or higher clinical RP groups. We chose imaging and dose datasets for patients with early‐stage lung cancer since these datasets are in general much cleaner (no pneumonia, atelectasis, or other pathologic changes) than images of patients with larger tumors. Also, radiation dose distributions are more localized and conformal, and post‐RT changes are easily identifiable. Despite its low incidence, RP is a serious side effect with potentially lethal outcomes in this population with typically severely compromised lung function. Often patients are already on supplemental oxygen or on oral steroids before treatment. RP grade 2 or higher, even if not lethal, can lead to further impairment of a patient's lung function, requiring initiation of oxygen supplement, reduced mobility, loss of work, need to go on disability, or limited ability to do activities of daily living.

This study applies a common deep learning approach used in non‐medical applications such as predicting image features from millions of 2D images to the medical imaging domain. Very few deep learning studies to date have explored stratification based on outcome parameters, with most studies exploring tasks such as image segmentation^32^ or malignancy detection.^33^ One of the many advantages of deep learning approaches is the automation of feature extraction whereas the traditional radiomics approaches have relied on complex manual feature extraction and selection techniques using shallower neural networks such as support vector machines and random forest algorithms.^34^ One of the major drawbacks of this approach is the dependence on the manually extracted engineered features where certain fine and minuscule imaging patterns may be neglected or missed and hence be unavailable for the machine learning models to be utilized for prediction purposes. The deep learning inputs are comprised of 3D voxel cubes that allow the network to consider not only the tumor volume but also the surrounding regions that, according to our study, appear to have high predictive power. More recently, deep learning approaches have become popular and are used as the de‐facto standard for machine learning on medical images.^35^, ^36^

Since our data is highly imbalanced (No RP vs. Yes RP), accuracy may not be the right measure to evaluate the performance of the models since they are based on how many samples, both positively and negatively, were correctly classified. Higher accuracy scores can be obtained by correctly classifying all the samples from the majority (No RP) class. Though class balancing approaches and data augmentation techniques have been utilized on the training dataset, the AUC and F1 scores on the test dataset were calculated by taking the harmonic mean between the precision and recall values. This provides a good measure to evaluate the model performance with imbalanced test datasets. In this study, we built 3D DenseNet and ResNet‐50 models of three (No RP, RP1, RP2) and two (No RP, Yes RP) classes as outputs. The Dense‐Net‐121 models performed better than the ResNet‐50 models with statistically significant results for the 3‐class prediction model. This could be due to the DenseNet‐121 model architecture where dense connections are established between all previous and subsequent layers and features learned in the top layers (e.g., ground glass opacity within the lung or consolidation changes in the lung parenchyma, etc.) with coarse convolution layers would also contribute to the eventual decision making for this classification. The three‐class prediction AUC_macro_ (0.91) was better than the two‐class prediction (0.84) for the DenseNet‐121 model. With ML approaches, the assumption is that effects of radiotherapy on ‐in our case ‐ lung tissue are complex and not deterministically defined. Useful models depend on the quality of input data to produce reasonable predictions. One thought is therefore that models learn better from well‐characterized feature classes that show obvious differences (by differentiating between RP1 and RP2), as opposed to creating a mixed feature class of RP1 and RP2 together. There could also be other factors that are inherently related to RP0, 1, and 2 that we are not aware of which nonetheless can lead to a clearer differentiation between RP0, 1, and 2 as opposed to RP0 versus RP1 and 2 combined. On the other hand, we cannot exclude that the number of samples from one of the two minority classes (RP1 or RP2) when concatenated might be randomly oversampled more than the other class. This would create more samples of one of the RP1 or RP2 classes in the combined Yes RP class used for the two class prediction models. This would be the reason why the two class models with mixed feature class of RP1 and RP2 would have a lower AUC than the three‐class model since the model would learn features for the class that has more input samples and not learn features for both classes in a balanced manner.

Our results using the images and dose patches as model inputs are consistent with published literature on deep learning‐based models for radiotherapy toxicity predictions. Ibragimov et al.^17^ reported that the AUC for a CNN‐based prediction model for hepatobiliary toxicities with liver SBRT cases was 0.79. They also reported that combining the CNNs with the 3D dose patched increased the AUC to 0.85. Zhen et al.^35^ also reported an AUC of 0.89 for the CNN models trained using transfer learning from a pre‐trained VGG16 model to predict rectum toxicities in cervical cancer radiotherapy. Su et al.^36^ investigated the use of an artificial neural network model with three fully connected feed‐forward networks using CT images of 142 patients treated with three‐dimensional conformal radiotherapy and achieved an AUC of 0.85. Others have used Support Vector Machines (SVM)^37^ (AUC = 0.72) or logistic regression^38^, ^39^ (AUC = 0.68) for these prediction models.

We also attempted to identify salient regions within the input 3D image dataset via an IG technique that provided important details of the tumor surrounding volume in the patient RP stratification. Based on our visual evaluation of IG maps with thirty patients (fifteen each for No RP and Yes RP) samples cases, these techniques appeared to indicate the significance of the tumor and the surrounding tissue in the prediction of lung injury for patients. We also found that voxels outside the tumor regions have contributed to the model predictions. Clinical verification of these findings is not possible based on the current understanding of the RP development process. Zhen et al.^35^ published deep learning models for the prediction of rectal toxicity. The saliency maps from this study indicated that the highly discriminative region for the predictions was the upper region of the rectum. It is more difficult to assess the predictions with IG in lung cancer patients than in rectum due to the much larger variability of anatomical topography in lung tumors. The clinical decision‐making based on these IG heat maps is challenging due to the fact that the highlighted regions seem to vary across various patient cases and are quite limited in their ability to explain the model behavior to clinical experts. Saporta et al.^40^ also found that the saliency methods for localization perform worse than expert localization across multiple analyses and many important pathologies. They also reported that when these maps are used in clinical practice, they can introduce well‐documented biases and erode the trust in model predictions, even when the model predictions are correct, thus limiting clinical applicability. Therefore, it is important that before these models are utilized in the clinical domain, they should be made more interpretable where detailed clinical interpretation of model predictions can be utilized to explain the salient features per output layers that are extracted and used for such toxicity predictions. We plan to build such interpretable models in our future work. At this point, while the findings on IG heat maps cannot be completely explained, showing the feasibility of generating IG maps and documenting the importance of the tumor and high dose area on these maps is promising.

One aspect we investigated was to study and build models learning from the temporal dimensions of these imaging datasets. There are recurrent neural networks (RNNs) that have the capacity to repeatedly learn based on previously remembered information for a time series‐based dataset and then apply that to the current dataset. The long short‐term memory‐based models are one type of RNN that is very commonly used to deal with time‐series based 2D datasets. Our challenge with such models has been the lack of multiple (more than 5) time points within our dataset. These techniques work well with video‐based datasets that have 20–30 2D frames at a minimum for the model to gather spatial and temporal changes in the image shape and appearance. We believe that once we have a sufficient number of follow‐up imaging datasets acquired on a consistent time‐series basis, we should be able to build a model that can quantify the subtle image changes over time and provide more meaningful clinical insights into radiological changes in lungs after radiation treatments.

Another weakness of this study is the limited‐size dataset compared to non‐medical applications. The dataset lacked more samples per region of tumor location for the algorithm to make generalizable predictions. Due to the heterogeneity, size, shape and location of the tumor regions, predicting the response to radiotherapy can be a difficult task. As a rule of thumb, deep learning models are usually trained using tens of thousands of samples. Although we have already observed good prediction with our moderate‐size cohort for the 3‐class model, these models can potentially benefit from a larger cohort of datasets with imaging studies at each follow‐up interval. Based on the experience from this study, as part of our future work, we, therefore, plan to also include locally advanced lung cancer patients which will in turn increase our sample size and variability of tumor morphology and dose distributions, but also increase the relative number of RP cases. This strategy will be good for CNN training because it will help balance the input training datasets.

In order to gather more data, data sharing must be encouraged between multiple treating institutions. However, multi‐institutional collaborations based on centrally shared patient data face privacy and ownership challenges. There are federated learning approaches^41^ that utilize novel concepts of model learning leveraging where none of the available data at an institution is shared but only the model learning is shared. These types of collaborative learning approaches use the same model to train on the dataset locally without sharing any dataset (with or without patient information) to a central repository but only sharing their model updates to the central repository. The aggregation central server receives model updates from multiple institutions and combines the model weights and then sends the consensus model to all collaborating institutions for use and/or further training. Some of the challenges with these approaches are enforcing uniformity in model architecture, utilizing standard computational processes, and providing adequate techniques for data quality and benchmarking before updating the input model weights on the central aggregation servers. However, this can be one of the methods for deep learning models to be trained with large datasets from multiple institutions.

Other than the limitations with the size of the dataset, it should be noted that the deep learning algorithms and their working mechanisms remain a black box. Of course, it is useful to have the imaging features automatically extracted based on the image patterns and textures, but there are problems with the implementation of these algorithms in clinical practice because physicians are not able to gather a clear understanding of how to intuitively interpret the results obtained by such models. Thus, one of the biggest challenges with deep learning approaches is determining the reasoning behind why and where certain characteristics in the input images have a positive or negative effect on the eventual predictions. Additionally, not having a balanced dataset with an equal number of RP and non‐RP patients does not help the training process where the model is seeing a greater number of non‐RP cases and learning very little about the RP cases' image patterns and features in order to provide generalizable predictions. With our study, we have balanced out the dataset by utilizing data augmentation techniques such as translation, rotation, etc. of the RP and non‐RP images before the training process. There are other oversampling techniques such as synthetic minority oversampling techniques (SMOTE)^42^ which generates new samples using the combination of nearby examples of the same class. These techniques are not guaranteed to generate realistic‐looking images or ones that are medically reasonable. We plan to study these techniques to make them applicable to medical imaging datasets and implement them in our future work.

In conclusion, we proposed a deep learning network to predict RP based on CT imaging scans and radiation treatment dose information. We demonstrated the model's ability to stratify patients in non‐radiation pneumonitis, grade 1 and grade 2, and higher pneumonitis groups. Consequently, we looked at regions in the input images that provide the most important information to guide the model with these predictions, thus narrowing the gap between computer science techniques used for pattern recognition and precision medicine. The clinical meaning of ROIs that are identified as important for the development of RP from these models needs further investigation.

## AUTHOR CONTRIBUTIONS

All authors have collectively contributed in data collection, analysis, preparation and editing of this manuscript.

## CONFLICT OF INTEREST

Dr. Elisabeth Weiss receives funding from NIH, is a member of Canon Medical Systems Speakers' bureau and receives royalties from UpToDate.
